# Supplementary material for: Correlative all-optical quantification of mass density and mechanics of subcellular compartments with fluorescence specificity
Source: eLife. 2022 Jan 10;11:e68490. doi: 10.7554/eLife.68490 (PMC8816383; doi:10.7554/eLife.68490)
Supplement: Supplementary file 1. [file elife-68490-supp1.docx]

**Supplementary Table 1.** Average values and standard errors of the mean of the RI *n*, Brillouin shift *ν*_B_, absolute density *ρ*, longitudinal modulus *M’* and linewidth Δ_B_ for the cytoplasm (cyto), nucleoplasm (np) and nucleoli (nl) of 139 wild-type HeLa cells.

| compartment | RI  *n* | Brillouin shift *ν*_B_ [GHz] | absolute density  *ρ* [g/ml] | longitudinal modulus  *M’* [GPa] | linewidth  Δ_B_ [GHz] |
| --- | --- | --- | --- | --- | --- |
| cytoplasm | 1*.*3545 ± 0*.*0004 | 7*.*811 ± 0*.*008 | 1*.*0234 ± 0*.*0006 | 2*.*410 ± 0*.*005 | 1*.*175 ± 0*.*008 |
| nucleoplasm | 1*.*3522 ± 0*.*0004 | 7*.*872 ± 0*.*007 | 1*.*0207 ± 0*.*0005 | 2*.*448 ± 0*.*005 | 1*.*193 ± 0*.*008 |
| nucleoli | 1*.*3618 ± 0*.*0004 | 7*.*938 ± 0*.*008 | 1*.*0310 ± 0*.*0005 | 2*.*487 ± 0*.*005 | 1*.*271 ± 0*.*010 |
