# Supplementary material for: Correlative all-optical quantification of mass density and mechanics of subcellular compartments with fluorescence specificity
Source: eLife. 2022 Jan 10;11:e68490. doi: 10.7554/eLife.68490 (PMC8816383; doi:10.7554/eLife.68490)
Supplement: Supplementary file 2. [file elife-68490-supp2.docx]

**Supplementary Table 2.** Kruskal-Walis $p$-values when comparing the RI $n$, Brillouin shifts $\nu_{\text{B}}$, mass densities $\rho$, longitudinal moduli $M^{'}$ and linewidths of the cytoplasm (cyto), nucleoplasm (np) and nucleoli (nl) of 139 wild-type HeLa cells, respectively.

| comparing | RI  $p_{{n_{c_{1}},n}_{c_{2}}}$ | Brillouin shift$p_{{\nu_{\text{B},c_{1}},\upsilon}_{{\text{B},c}_{2}}}$ | absolute density lo  $p_{{\rho_{c_{1}},\rho}_{c_{2}}}$ | ongitudinal modulus  $p_{{M^{'}}_{c_{1}},{M^{'}}_{c_{2}}}$ | linewidth $p_{{\Delta_{\text{B},c_{1}},\Delta}_{{\text{B},c}_{2}}}$ |
| --- | --- | --- | --- | --- | --- |
| *c*_1_:cyto to *c*_2_:np | 9 × 10−4 | 2 × 10−6 | 3 × 10−3 | 7 × 10−7 | 0.139 |
| *c*_1_:cyto to *c*_2_:nl | 1 × 10−21 | 1 × 10−23 | 7 × 10−17 | 4 × 10−23 | 8 × 10−14 |
| *c*_1_:np to *c*_2_:nl | 2 × 10−37 | 2 × 10−7 | 3 × 10−29 | 9 × 10−7 | 2 × 10−8 |
