# Supplementary material for: Correlative all-optical quantification of mass density and mechanics of subcellular compartments with fluorescence specificity
Source: eLife. 2022 Jan 10;11:e68490. doi: 10.7554/eLife.68490 (PMC8816383; doi:10.7554/eLife.68490)
Supplement: Supplementary file 3. [file elife-68490-supp3.docx]

**Supplementary Table 3.** Average values and standard errors of the mean of the RI $n$, Brillouin shift $\nu_{B}$, absolute density $\rho$ and longitudinal modulus $M^{'}$ for the cytoplasm and polyQ aggregates of 22 wild-type HeLa cells.

| compartment | RI  *n* | Brillouin shift  *ν*_B_ [GHz] | absolute density  *ρ* [g/ml] | longitudinal modulus  *M’* [GPa] |
| --- | --- | --- | --- | --- |
| cytoplasm | 1*.*3506 ± 0*.*0013 | 7*.*861 ± 0*.*014 | 1*.*020 ± 0*.*002 | 2*.*442 ± 0*.*009 |
| polyQ aggregate | 1*.*3856 ± 0*.*0018 | 8*.*789 ± 0*.*040 | 1*.*061 ± 0*.*003 | 3*.*051 ± 0*.*029 |
