# Supplementary material for: Correlative all-optical quantification of mass density and mechanics of subcellular compartments with fluorescence specificity
Source: eLife. 2022 Jan 10;11:e68490. doi: 10.7554/eLife.68490 (PMC8816383; doi:10.7554/eLife.68490)
Supplement: Supplementary file 4. [file elife-68490-supp4.docx]

**Supplementary Table 4.** Average values and standard errors of the mean of the RI $n$ and longitudinal modulus $M^{'}$ for different conditions and compartments of P525L HeLa cells.

| condition | compartment | RI  $n$ | longitudinal modulus $M^{'}$ [GPa] |
| --- | --- | --- | --- |
| control | cytoplasm | 1*.*3481 ± 0*.*0010 | 2*.*361 ± 0*.*009 |
|  | nucleoplasm | 1*.*3509 ± 0*.*0008 | 2*.*409 ± 0*.*006 |
| arsenite | peri-SG  cytoplasm | 1*.*3456 ± 0*.*0007 | 2*.*329 ± 0*.*009 |
|  | nucleoplasm | 1*.*3476 ± 0*.*0006 | 2*.*394 ± 0*.*009 |
|  | SGs | 1*.*3443 ± 0*.*0006 | 2*.*345 ± 0*.*009 |
| arsenite, fixed | peri-SG  cytoplasm | 1*.*3443 ± 0*.*0005 | 2*.*331 ± 0*.*006 |
|  | nucleoplasm | 1*.*3485 ± 0*.*0005 | 2*.*416 ± 0*.*006 |
|  | SGs | 1*.*3454 ± 0*.*0004 | 2*.*357 ± 0*.*006 |
